# The Risk Factors for Myopia Undercorrection in Second-Generation (Visumax 800) Keratorefractive Lenticule Extraction Surgery: A Retrospective Case–Control Study

**DOI:** 10.3390/diagnostics14161752

**Published:** 2024-08-12

**Authors:** Chia-Yi Lee, Shun-Fa Yang, Hung-Chi Chen, Ie-Bin Lian, Chin-Te Huang, Jing-Yang Huang, Chao-Kai Chang

**Affiliations:** 1 Institute of Medicine, Chung Shan Medical University, Taichung 40201, Taiwan; 2 Nobel Eye Institute, Taipei 10041, Taiwan; 3 Department of Ophthalmology, Jen-Ai Hospital Dali Branch, Taichung 41265, Taiwan; 4 Department of Medical Research, Chung Shan Medical University Hospital, Taichung 40201, Taiwan; 5 Department of Ophthalmology, Chang Gung Memorial Hospital, Taoyuan 33305, Taiwan; mr3756@cgmh.org.tw; 6 Center for Tissue Engineering, Chang Gung Memorial Hospital, Taoyuan 33305, Taiwan; 7 Department of Medicine, Chang Gung University College of Medicine, Taoyuan 33305, Taiwan; 8 Institute of Statistical and Information Science, National Changhua University of Education, Chunghua 50007, Taiwan; 9 Department of Ophthalmology, Chung Shan Medical University Hospital, Taichung 40201, Taiwan; 10 Department of Ophthalmology, School of Medicine, Chung Shan Medical University, Taichung 40201, Taiwan; 11 Department of Optometry, Da-Yeh University, Chunghua 51591, Taiwan

**Keywords:** keratorefractive lenticule extraction, visumax 800, smile pro, uncorrected distance visual acuity, myopia

## Abstract

In this study, we aim to evaluate the risk factors of myopia undercorrection in recipients of second-generation keratorefractive lenticule extraction (KLEx) surgery. A retrospective case–control study was performed, and patients who received second-generation KLEx surgery were enrolled. The cases with myopia undercorrection were matched to non-myopia undercorrection cases with a 1:4 ratio according to age, and a total of 22 and 88 eyes were categorized into the undercorrection and control groups, respectively. Demographic, refractive, topographic, and surgical data were collected preoperatively. A generalized linear model was operated to evaluate the potential risk factors for myopia undercorrection. The uncorrected distance visual acuity (UDVA) at three months postoperation was significantly better in the control group (*p* = 0.006), and residual myopia and SE were significantly higher in the undercorrection group during the whole follow-up period (all *p* < 0.001). The UDVA value showed a trend of improvement in the control group (*p* < 0.001), and the changes to SE and residual myopia were significantly lower in the control group (both *p* < 0.001). Regarding the risk factors for myopia undercorrection in the whole population and the high-myopia population, a higher manifest sphere power, higher steep keratometry (K), higher topographic cylinder, lower central corneal thickness (CCT) at apex, higher CCT difference and lower residual stromal thickness (RST) correlated to myopia undercorrection (all *p* < 0.05). In the low-myopia population, only higher myopia and lower RST correlated to myopia undercorrection (both *p* < 0.05). In conclusion, a high-sphere power and irregular topographic pattern correlated to myopia undercorrection after the second KLEx surgery, especially for individuals with high myopia.

## 1. Introduction

Keratorefractive surgery refers to a method for myopia, hyperopia, and astigmatism correction via the removal of the corneal tissue [1,2]. Laser in situ keratomileusis and photorefractive keratectomy have been in use for more than 15 years, and the visual and refractive outcomes of these keratorefractive surgeries have been fair [2]. More than 65 percent of individuals received laser in situ keratomileusis and a photorefractive keratectomy uncorrected distance visual acuity (UDVA) of 20/20 [3,4]. On the other hand, postoperative complications, including ocular irritation, postoperative dry eye disease, and myopia regression, have been reported following these keratorefractive surgeries [5,6].

Keratorefractive lenticule extraction (KLEx) [7], known by the brand name of small incision lenticule extraction, is a keratorefractive surgery that was introduced about 14 years ago [7,8,9,10]. In comparison with laser in situ keratomileusis and photorefractive keratectomy, KLEx has the advantage of a small incision, which results in fewer postoperative cases of dry eye disease and ocular irritation [11,12]. Regarding surgical outcomes, first-generation KLEx was found to be consistent not only for laser in situ keratomileusis but also for photorefractive keratectomy, based on earlier publications [13,14,15,16]. In addition, the amount of postoperative astigmatism was comparable between first-generation KLEx surgery and wavefront-guided laser in situ keratomileusis [17,18]. Nevertheless, myopia regression and undercorrection could be developed following first-generation KLEx surgery, both with the risk factors of a high initial myopia degree and steep keratometry (K) [19,20,21].

The second generation of KLEx surgery was applied in clinical practice in the year 2023 [22]. Second-generation KLEx has the advantage of a lower laser strike period and eye-tracking system equipment compared to first-generation KLEx surgery [23,24]. Still, there has been no study to evaluate the risk factor for myopia undercorrection in second-generation KLEx surgery. Because of the differences in instruments and programs between first- and second-generation surgeries, second-generation KLEx surgery may have different risk factors for myopia undercorrection.

As a result, the aim of our study is to explore the risk factors for myopia undercorrection in second-generation KLEx surgery. The risk factors in high- and low-myopia populations were checked separately.

## 2. Materials and Methods

### 2.1. Participant Enrollment

This retrospective case–control study was carried out at the Nobel Eye Institute, which has multiple branches in the northern, central, and southern regions of Taiwan. Patients were enrolled in the study population with the following inclusion criteria: (1) aged 20–55 years; (2) cycloplegia sphere power from −1.00 diopter (D) to −9.00D; (3) cycloplegia cylinder power lower than −5.00D; (4) received second-generation KLEx surgery at any clinic of the Nobel Eye Institute; and (5) followed up in any Nobel Eye institute branch after the second KLEx surgery for at least three months. If a patient underwent KLEx surgery with monovision (planning residual myopia), the patient was excluded from our study. In addition to the inclusion criteria, consecutive exclusion criteria were utilized to remove patients with prominent impaired ophthalmic status: (1) a best-corrected visual acuity (BCVA) lower than 20/40; (2) the presence of severe ocular diseases before second-generation KLEx surgery, including but not limited to central corneal opacity, uncontrolled glaucoma, proliferative diabetic retinopathy, advanced uveitis, keratoconus, central retinal venous occlusion and macula-off retinal detachment; (3) a refraction fluctuation of more than 0.50D of sphere power during the last year; and (4) undergoing pregnancy or breastfeeding during the last three months. Then, patients with a residual sphere power of more than −1.00D at the three-month follow-up and a change in sphere power of more than −0.25D during the follow-up period were regarded as the undercorrection group. The eye to be enrolled in our study was decided by drawing lots, and then one eye with myopia undercorrection was matched to four eyes without myopia undercorrection via age (within 5 years) with a 1:4 ratio of eye numbers. Finally, a total of 22 and 88 eyes were categorized into the undercorrection group and control group, respectively.

### 2.2. Surgery Technique

The second-generation KLEx surgery in our study was performed by two experienced refractive specialists (i.e., C.-Y.L. and C.-K.C.). After deciding the target refraction according to the manifest refraction and cycloplegia refraction, the surgical nomogram of myopia in second-generation KLEx surgery was (1) target refraction × 1.1 for those with myopia lower than −2.50D, (2) (target refraction plus −0.15D) × 1.1 for those with myopia from −2.50D to −5.00D, (3) (target refraction plus −0.25D) × 1.1 for those with myopia from −5.00D to −7.50D, and (4) (target refraction plus −0.40D) × 1.1 for those with myopia more than −0.75D. Regarding astigmatism, the surgical nomogram in second-generation KLEx surgery is directly related to the target refraction of astigmatism, which is based on manifest refraction and cycloplegia refraction. It is carried out with one second-generation femtosecond laser device (Visumax 800, Carl Zeiss, Göschwitzer Str., Jena, Germany). The optic zone is settled from 5.5 to 6.9 mm, considering the ablation depth and pupil size, and the corneal incision is set to 3.0 mm at 105 degrees. After the angle kappa is defined by a microscope with topographical assistance and the coaxial-sighted corneal light reflex approach, the corneal surface is settled in a suction ring, and the angle kappa defined by optical biometry (IOL Master 700, Carl Zeiss, Göschwitzer Str., Jena, Germany) is shown on a monitor via the Visumax 800 for further assistance. After 8–10 s of femtosecond laser emission, a spatula is applied to disconnect the superior and inferior interface of the corneal lenticule, and then the corneal lenticule is taken off. Levofloxacin and prednisolone eye drops are administered postoperatively for approximately one week, and then sulfamethoxazole and fluorometholone eye drops are administered for three weeks. Artificial tears are administered for at least two months after second-generation KLEx surgery.

### 2.3. Ophthalmic Examination

The ophthalmic examinations in patients who had undergone second-generation KLEx surgery were identical in all clinics of the Nobel Eye Institute. The preoperative exams included BCVA via manifest refraction and cyclopegia refraction with an autorefractor (KR-8900, Topcon, Itabashi-ku, Tokyo, Japan). The steep and flat K, central corneal thickness (CCT) at the apex and thinnest region, corneal astigmatism, angle kappa, and pupil diameter were checked using a topographic machine (TMS-5, Tomey Corporation, Nagoya, Aichi, Japan). An additional angle kappa was yielded with an abiometry machine (IOL Master 700, Carl Zeiss, Göschwitzer Str., Jena, Germany). After the second-generation KLEx surgery, the UDVA, manifest sphere power, and manifest cylinder power were examined. The postoperative exams were performed using the same devices and techniques as in the preoperative exams. The surgical parameters, including optic zone (OZ), cap thickness, side-cut depth, residual stromal thickness (RST), and lenticular thickness, were recorded from the surgical notes. Exams were taken before surgery, one day postoperatively, one week postoperatively, one month postoperatively and three months postoperatively in all patients. In addition, the best-corrected visual acuity (BCVA) three months postoperatively, in the two groups was also obtained. The spherical equivalent (SE) was delineated as the sphere power plus the half-cylinder power in our study, and the angle kappa value was delineated as the average value of the angle kappa from the topographic instrument and biometry instrument. The CCT difference was delineated as the CCT value at the apex minus the CCT value at its thinnest, and the angle kappa difference was delineated as the angle kappa obtained by biometry minus the angle kappa obtained by topography.

### 2.4. Statistical Analysis

SPSS version 20.0 (SPSS Inc., Chicago, IL, USA) was used for the statistical analysis mentioned in our study. The Shapiro–Wilk test was employed to check the normality of the whole population, and a normal distribution was found (*p* > 0.05). Descriptive analysis was utilized to demonstrate the age, sex, manifest, and cycloplegia refractions, topographic details, and surgical details of the two groups, and then an independent T test was performed to check the differences in these indices between the two groups. The independent T test was also used to check the efficiency (i.e., UDVA), predictability (i.e., SE), and residual sphere powers between the undercorrection and control groups after second-generation KLEx surgery. Line charts with the standard error of the mean were created to illustrate the trends of UDVA, SE, and sphere power changes between the two groups, and the generalized estimate equation was utilized to check the differences in these trends between groups with the production of the adjusted odds ratio (aOR) and 95% confidence interval (CI), which was adjusted for the effects of age and sex. In the next step, the generalized linear model was used to examine the potential preoperative risk factor for myopia undercorrection three months postoperatively in the whole population. Then, the whole study population was separated into those with low myopia (less than −6.00D preoperative cycloplegia SE) and high myopia (more than −6.00D preoperative cycloplegia SE), and the potential risk factors for myopia undercorrection were checked using the generalized linear model in both subgroups. The aOR and 95% CI were also produced in the generalized linear model analysis. A *p* value lower than 0.05 was specified as showing statistical significance.

## 3. Results

The basic characteristics of the two groups are presented in Table 1. The mean ages were 34.27 ± 6.03 and 32.00 ± 8.50 in the undercorrection and control groups, respectively (*p* = 0.149). Additionally, the distribution of sex, systemic diseases, and laterality was statistically similar between the two groups (all *p* > 0.05). Regarding the ophthalmic parameters, the BCVA between the two groups was statistically identical (*p* = 0.079), and the manifest and cycloplegia sphere powers showed a non-significantly higher value in the undercorrection group than in the control group (both *p* > 0.05). The rest of the ophthalmic parameters did not demonstrate significant differences between the two groups (all *p* > 0.05) (Table 1).

**Figure 1:**
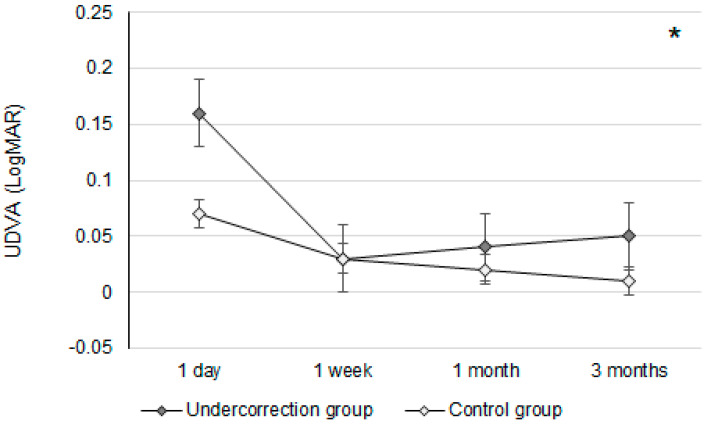
The trend of uncorrected distance visual acuity changes in the two groups. UDVA: uncorrected distance visual acuity; * denotes significant differences in the trends of uncorrected distance visual acuity changes between the two groups (*p* < 0.05).

**Figure 2:**
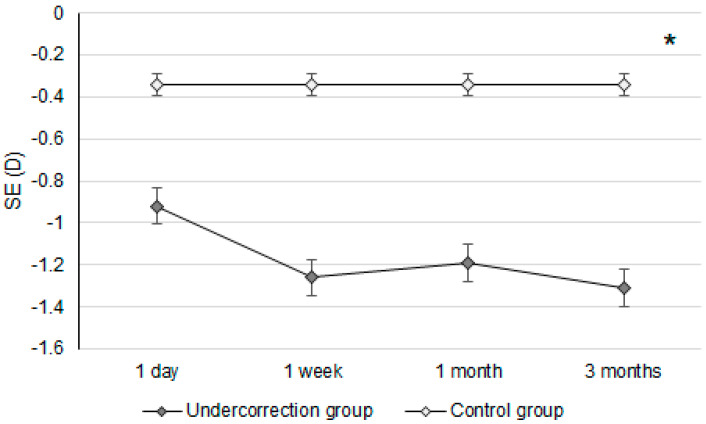
The trend of spherical equivalent changes in the two groups. D: diopter and SE: spherical equivalent; * denotes significant differences in the trends of spherical equivalent changes between the two groups (*p* < 0.05).

**Figure 3:**
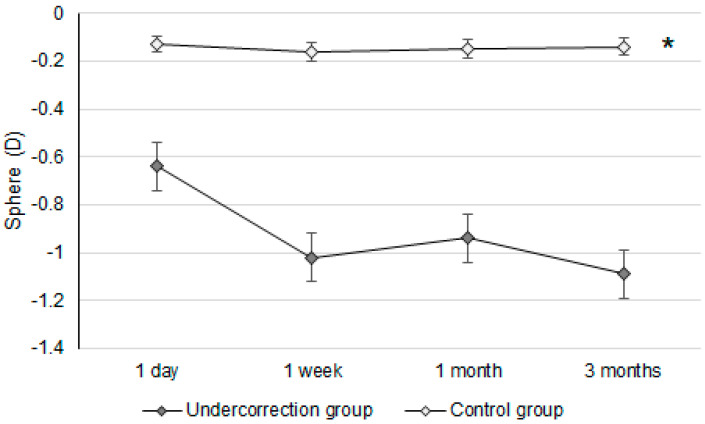
The trend of sphere power changes in the two groups. D: diopter; * denotes significant differences in the trends of sphere power changes between the two groups (*p* < 0.05).

Initially, the UDVA was similar between the two groups (*p* = 0.095). However, the UDVA three months postoperatively was significantly better in the control group than in the undercorrection group (*p* = 0.006) (Table 2). The BCVA three months postoperatively was 0.00 ± 0.02 in the undercorrection group and 0.00 ± 0.01 in the control group without a significant difference (*p* = 0.998). On the other hand, residual myopia and SE were significantly higher in the undercorrection group than those in the control group from postoperative day one to the final visit (all *p* < 0.001) (Table 2). Concerning the changes in postoperative outcomes, the UDVA value showed a trend of decreasing (i.e., better visual acuity) in the control group (aOR: 0.762, 95% CI: 0.426–0.853, *p* < 0.001) (Figure 1). In addition, the changes in SE (aOR: 0.621, 95% CI: 0.312–0.788, *p* < 0.001) and residual myopia (aOR: 0.555, 95% CI: 0.478–0.645, *p* < 0.001) were significantly lower in the control group (Figure 2 and Figure 3).

Regarding the risk factors for undercorrection in the whole population, a higher manifest sphere power, higher steep K, higher topographic cylinder, lower CCT at apex, higher CCT difference, and lower RST correlated to undercorrection (all *p* < 0.05) (Table 3). In the high-myopia population, a higher manifest sphere power, higher steep K, higher topographic cylinder, lower CCT at apex, higher CCT difference, and lower RST correlated to undercorrection (all *p* < 0.05) (Table 4). In the low-myopia population, only the higher manifest sphere power and lower RST correlated to undercorrection (both *p* < 0.05) (Table 5).

## 4. Discussion

In our study, myopia undercorrection after second-generation KLEx surgery led to worse postoperative outcomes. In addition, higher preoperative myopia, higher corneal refractive power variance, and lower RST correlated to myopia undercorrection, especially in the high-myopia population.

The UDVA differences between the undercorrection and control groups were not significant until three months after the second-generation KLEx surgery. In earlier studies, which surveyed postoperative vision following second-generation KLEx surgery, more than 90 percent of participants reached 20/20 UDVA three months after second-generation KLEx surgery [23]. Nevertheless, no research has been performed to evaluate the postoperative UDVA between myopia undercorrection and non-myopia undercorrection populations who received second-generation KLEx surgery. To our knowledge, the findings of this study may be relatively new in demonstrating the lower UDVA of myopia undercorrection after second-generation KLEx surgery with an adequate follow-up period. The baseline characteristics between the undercorrection and control groups were statistically identical, and thus, the homogeneity of the study population is acceptable. Although the manifest and cycloplegia sphere powers were numerically higher in the undercorrection group, the difference in sphere power between the two groups was around −0.60D, which might not have a significant influence. Concerning the UDVA between the undercorrection and control groups at different time points, the UDVAs at one-day postoperation were relatively poor in both groups, which may have been a consequence of the higher laser frequency and greater laser emission in second-generation KLEx surgery [23,24]; therefore, postoperative corneal edema may be prominent. On the other hand, the UDVA kept improving in the control group throughout the postoperative follow-up period, while the undercorrection group showed a progressively worse UDVA from one-week postoperation. However, the absolute amount of UDVA differences between the two groups (0.04 LogMAR) indicates that myopia undercorrection could influence the UDVA in just three months. On the other hand, the BCVA three months postoperatively between the two groups was nearly identical, which indicates that the worse UDVA in the undercorrection group may have resulted from undercorrection rather than corneal ectasia.

Regarding postoperative refraction between the undercorrection and control groups, postoperative SEs and residual myopia demonstrated a higher value in the undercorrection group than in the control group. The postoperative SE following first-generation KLEx surgery was around −0.10 to −0.20D [25], and more than 80 percent of participants reached fine refraction within ±0.50D three months after second-generation KLEx surgery [23]. The control group in our study presented a similar predictability of SE compared to previous publications [23,25]. In addition, the postoperative residual myopia in our control group was also not inferior to previous studies discussing KLEx surgeries [23,26]. On the other hand, postoperative SE and residual myopia were significantly higher in the undercorrection group throughout the study period compared to the control group and an earlier publication that evaluated second-generation KLEx surgery [23]. The change in both SE and residual myopia in the undercorrection group showed a significant trend towards increment compared to the control group, and the mean residual myopia was about −0.64D one day postoperation in the undercorrection group, increasing to −1.09D three months postoperation in the undercorrection group. The trend of SE in the undercorrection group also showed a tendency to increase, which was similar to the trend of residual myopia, indicating that the astigmatism amount did not correlate to astigmatism undercorrection in our study in conflict with previous results that used first-generation KLEx surgery [27]. A possible reason for this is that second-generation KLEx surgery uses an eye-tracking system that may manage astigmatism better than the previous version [23,24]. Still, further studies are needed to verify the etiology of discordance between postoperative myopia and astigmatism changes.

The risk factors for myopia undercorrection in the patients who received second-generation KLEx surgery included higher preoperative myopia, higher steep K, higher corneal astigmatism, a lower CCT apex value, higher CCT difference, and lower RST. There has been little research indicating the risk factors for myopia undercorrection following second-generation KLEx surgery. In previous studies that evaluated the risk factors of undercorrection for first-generation KLEx (Visumax 500) surgery, the higher preoperative myopia and higher K value correlated with myopia undercorrection [20,28,29]. Regarding other refractive surgeries, higher preoperative manifested refractive power, including myopia and astigmatism and lower CCT, was associated with the development of myopia undercorrection of laser in situ keratomileusis [30], and enhancement was commonly applied to manage the undercorrection of in laser in situ keratomileusis [31,32]. On the other hand, higher preoperative SE and smaller OZ was positively associated with the undercorrection of photorefractive keratectomy in earlier studies in the literature [33]. Considering the studies demonstrated above, our findings may suggest that high preoperative refractive power is a universal risk factor for undercorrection in all keratorefractive surgeries, while steep corneal curvature is an additional risk factor for undercorrection in KLEx surgeries. The early postoperative CCT difference has also been proposed as a risk factor for early myopia regression following first-generation KLEx surgery [34], and our study additionally indicated the importance of preoperative CCT differences for myopia undercorrection in second-generation KLEx surgery as well. On the other hand, the angle kappa value and the difference between the topographic angle kappa and biometric angle kappa did not relate to myopia undercorrection. A large angle kappa was correlated to a high residual refractive error in the first KLEx surgery [35], and the eye-tracking system in second-generation KLEx surgery could allow the centration process to become more precise, thus reducing the influence of the angle kappa. In the subgroup analysis, the higher myopia subgroup had identical risk factors for myopia undercorrection compared to the whole group, while only a higher preoperative myopia degree and lower RST were associated with myopia undercorrection in the low-myopia group. This result may confirm the major role of high myopia levels in myopia undercorrection after second-generation KLEx surgery and the necessity to adjust the correction method in this population.

Regarding the postoperative efficiency and predictability between second-generation KLEx surgery in our study and different keratorefractive surgeries in other articles, the mean UDVA of second-generation KLEx surgery in the control group was 0.01 at three months postoperatively, which resembles the results of the UDVA after laser in situ keratomileusis and photorefractive keratectomy in other studies [4,36]. When it comes to predictability, about 91.3 percent of participants in the control group demonstrated a postoperative SE within ±1.00D after three months, which also resembles the predictability of first-generation KLEx surgery or laser in situ keratomileusis in other studies [25,36]. Moreover, the prevalence of residual myopia in the control group was also compatible with the residual myopia in the above two studies [25,36]. If we compare the efficiency and predictability of second-generation KLEx surgery in our study to previous research, which used wavefront-guided laser in situ keratomileusis, the UDVA and SE are similar to previous experience [37,38]. However, the postoperative outcomes of the undercorrection group in our study were numerically worse than in previous research [4,25,36]. Comparing the postoperative outcomes between the control group in our study and previous studies discussing second-generation KLEx surgery, the mean postoperative UDVA was −0.02, and 100 percent of participants reached a postoperative SE within ±1.00D [39]. Our results may be numerically inferior, but not clinically inferior, compared to previous experience [39]. The above results may suggest that the performance of second-generation KLEx surgery in our institution is not inferior to the keratorefractive surgeries reported in other institutions. Additionally, the prominent difference in postoperative outcomes between the control and undercorrection groups indicates the importance of detecting possible risk factors for myopia undercorrection in second-generation KLEx surgery.

There are a few limitations to our study. Firstly, the retrospective nature of our study could reduce the homogeneity of the study population, although no preoperative parameters showed a significant difference between the control and undercorrection groups. In addition, the numbers of eyes in the undercorrection and control groups illustrated a significant difference; the eye numbers in the control group had to be four times higher than the undercorrection group to reach acceptable statistical power. This discordance in eye numbers may, therefore, contribute to some statistical bias. Additionally, the postoperative topographic parameters were not recorded and analyzed due to the retrospective design of our study (we did not routinely measure topography after the second KLEx surgery or other refractive surgeries). Thus, the longitudinal analysis of topographic factors could not be conducted, and the postoperative changes in topographic parameters remain unknown. Also, epithelial cell thickness was not measured in our study. These shortcomings could affect the integrity of our results and conclusions to a huge extent. Finally, all the participants in our study were Taiwanese, so the external validity of our study could be reduced.

## 5. Conclusions

In conclusion, a steep, discordant, and thin topographic pattern and high preoperative myopia correlated to myopia undercorrection following second-generation KLEx surgery. Furthermore, the high-myopia population experienced more risk factors for myopia undercorrection compared to the low-myopia population. Consequently, the possibility of myopia undercorrection and enhancement should be communicated to individuals scheduled for second-generation KLEx surgery, along with the multiple risk factors involved. Further large-scale prospective studies are needed to evaluate the optimal correction method for reducing myopia undercorrection in second-generation KLEx surgery.

## Figures and Tables

**Table 1:** The baseline features of the study population.

Feature	Undercorrection Group (N: 22)	Control Group (N: 88)	*p*
Age	34.27 ± 6.03	32.00 ± 8.50	0.149
Sex (male/female)	6:16	43:55	0.230
Laterality (right/left)	10:12	63:35	0.146
Disease			0.369
Hypertension	0	5	
Diabetes mellitus	1	1	
Other	1	6	
BCVA (LogMAR)	0.00 ± 0.00	0.00 ± 0.02	0.079
Manifest refraction			
Sphere	−5.40 ± 1.62	−4.82 ± 1.85	0.178
Cylinder	−1.16 ± 0.95	−0.94 ± 0.95	0.351
SE	−5.98 ± 1.84	−5.29 ± 2.07	0.153
Cycloplegia refraction			
Sphere	−5.28 ± 1.66	−4.64 ± 2.10	0.184
Cylinder	−1.18 ± 0.99	−1.01 ± 0.95	0.435
SE	−5.88 ± 1.81	−5.14 ± 2.33	0.058
Topography			
Steep K	43.37 ± 2.22	43.84 ± 1.60	0.362
Flat K	41.99 ± 1.91	42.49 ± 1.37	0.253
Cylinder power	1.38 ± 0.82	1.33 ± 0.70	0.755
CCT at apex	550.36 ± 49.25	551.95 ± 31.87	0.886
CCT at thinnest	544.32 ± 48.94	546.12 ± 31.73	0.870
CCT difference	6.05 ± 2.66	5.83 ± 3.46	0.781
Angle kappa	0.20 ± 0.07	0.18 ± 0.09	0.422
Angle kappa difference	0.02 ± 0.07	0.04 ± 0.08	0.421
Pupil diameter	3.88 ± 0.60	3.71 ± 0.59	0.261
Schirmer test	13.09 ± 7.18	15.68 ± 7.63	0.149
Optic zone	6.55 ± 0.21	6.55 ± 0.13	0.907
Side-cut depth	14.32 ± 4.44	16.97 ± 6.26	0.062
Cap diameter	7.55 ± 0.21	7.54 ± 0.13	0.839
Cap thickness	110.23 ± 8.52	115.20 ± 6.85	0.528
RST	305.05 ± 24.60	317.51 ± 31.38	0.146
Lenticule thickness	131.91 ± 31.22	117.32 ± 30.14	0.081

BCVA: best-corrected visual acuity, CCT: central corneal thickness, N: number, RST: residual stromal thickness, and SE: spherical equivalent.

**Table 2:** Postoperative visual and refractive conditions between the two groups.

Outcome	Undercorrection Group (N: 22)	Control Group (N: 88)	*p*
UDVA			
1 day	0.16 ± 0.19	0.07 ± 0.09	0.095
1 week	0.03 ± 0.08	0.03 ± 0.06	0.792
1 month	0.04 ± 0.05	0.02 ± 0.05	0.092
3 months	0.05 ± 0.06	0.01 ± 0.04	0.006 *
SE			
1 day	−0.92 ± 0.57	−0.34 ± 0.52	<0.001 *
1 week	−1.26 ± 0.67	−0.34 ± 0.46	<0.001 *
1 month	−1.19 ± 0.57	−0.34 ± 0.41	<0.001 *
3 months	−1.33 ± 0.40	−0.33 ± 0.37	<0.001 *
Sphere			
1 day	−0.64 ± 0.64	−0.13 ± 0.53	<0.001 *
1 week	−1.02 ± 0.71	−0.16 ± 0.47	<0.001 *
1 month	−0.94 ± 0.63	−0.15 ± 0.43	<0.001 *
3 months	−1.09 ± 0.47	−0.14 ± 0.39	<0.001 *

N: number, SE: spherical equivalent, and UDVA: uncorrected distance visual acuity; * denotes a significant difference between groups.

**Table 3:** The risk factors for undercorrection in the whole population.

Factor	aOR	95% CI		*p*
		Lower	Upper	
Group	1.372	1.313	1.441	<0.001 *
Manifest sphere	1.127	1.024	1.240	0.014 *
Cycloplegia sphere	0.964	0.895	1.038	0.331
Steep K	1.992	1.011	2.925	0.046 *
Flat K	0.504	0.253	1.002	0.051
Topographic cylinder	1.408	1.210	1.793	0.008 *
CCT at apex	0.977	0.958	0.996	0.019 *
CCT at thinnest	1.030	0.919	1.051	0.085
CCT difference	1.132	1.078	1.647	0.001 *
Angle kappa	0.664	0.296	1.487	0.319
Angle kappa difference	1.142	0.472	2.766	0.768
OZ	1.119	0.688	1.820	0.650
RST	0.992	0.988	0.997	0.001 *

aOR: adjusted odds ratio, CCT: central corneal thickness, CI: confidence interval, K: keratometry, OZ: optic zone, and RST: residual stromal thickness; * denotes significant correlation to undercorrection. The risk factors are changes in OR per unit using a continuous value.

**Table 4:** The risk factors for undercorrection in the high-myopia population.

Factor	aOR	95% CI		*p*
		Lower	Upper	
Group	1.453	1.370	1.555	<0.001 *
Manifest sphere	1.275	1.134	1.576	0.009 *
Cycloplegia sphere	0.976	0.665	1.432	0.899
Steep K	1.714	1.098	3.272	0.002 *
Flat K	0.614	0.320	1.179	0.143
Topographic cylinder	0.429	0.232	0.795	0.007 *
CCT at apex	0.974	0.954	0.994	0.012 *
CCT at thinnest	1.021	0.998	1.045	0.075
CCT difference	1.564	1.213	2.464	0.015 *
Angle kappa	1.000	0.274	3.644	0.998
Angle kappa difference	0.800	0.147	4.364	0.797
OZ	1.084	0.247	2.626	0.150
RST	0.995	0.985	0.998	0.011 *

aOR: adjusted odds ratio, CCT: central corneal thickness, CI: confidence interval, K: keratometry, OZ: optic zone, and RST: residual stromal thickness; * denotes significant correlation to undercorrection. The risk factors are changes in OR per unit using a continuous value.

**Table 5:** The risk factors for undercorrection in the low-myopia population.

Factor	aOR	95% CI		*p*
		Lower	Upper	
Group	1.334	1.263	1.425	<0.001
Manifest sphere	1.136	1.018	1.268	0.023 *
Cycloplegia sphere	0.969	0.898	1.046	0.420
Steep K	1.433	0.150	3.661	0.755
Flat K	0.663	0.069	6.368	0.722
Topographic cylinder	0.609	0.063	5.862	0.668
CCT at apex	0.991	0.954	1.030	0.661
CCT at thinnest	1.016	0.977	1.057	0.432
CCT difference	1.009	0.923	1.268	0.061
Angle kappa	0.436	0.157	1.209	0.111
Angle kappa difference	1.668	0.620	4.488	0.311
OZ	0.920	0.495	1.711	0.793
RST	0.992	0.986	0.997	0.004 *

aOR: adjusted odds ratio, CCT: central corneal thickness, CI: confidence interval, K: keratometry, OZ: optic zone, and RST: residual stromal thickness; * denotes significant correlation to undercorrection. The risk factors are changes in OR per unit using a continuous value.

## Data Availability

The data used in our study are available from the corresponding author upon reasonable request.

## References

[1] Ganesh S., Brar S., Arra R.R. (2018). Refractive lenticule extraction small incision lenticule extraction: A new refractive surgery paradigm. Indian J. Ophthalmol..

[2] Ang M., Gatinel D., Reinstein D.Z., Mertens E., Alió Del Barrio J.L., Alió J.L. (2021). Refractive surgery beyond 2020. Eye.

[3] Padmanabhan P., Mrochen M., Basuthkar S., Viswanathan D., Joseph R. (2008). Wavefront-guided versus wavefront-optimized laser in situ keratomileusis: Contralateral comparative study. J. Cataract. Refract. Surg..

[4] Abdel-Radi M., Shehata M., Mostafa M.M., Aly M.O.M. (2023). Transepithelial photorefractive keratectomy: A prospective randomized comparative study between the two-step and the single-step techniques. Eye.

[5] Chang J.Y., Lin P.Y., Hsu C.C., Liu C.J. (2022). Comparison of clinical outcomes of LASIK, Trans-PRK, and SMILE for correction of myopia. J. Chin. Med. Assoc..

[6] Zhang Z., Xiang L.X., Wu Y., Li Q., Ke S.H., Liu L.Q. (2024). Factors affecting long-term myopic regression after corneal refractive surgery for civilian pilots in southwest China. BMC Ophthalmol..

[7] Dupps W.J., Randleman J.B., Kohnen T., Srinivasan S., Werner L. (2023). Scientific Nomenclature for Keratorefractive Lenticule Extraction (KLEx) Procedures: A Joint Editorial Statement. J. Refract. Surg..

[8] Reinstein D.Z., Carp G.I., Archer T.J., Vida R.S., Yammouni R. (2022). Large Population Outcomes of Small Incision Lenticule Extraction in Young Myopic Patients. J. Refract. Surg..

[9] Reinstein D.Z., Archer T.J., Vida R.S., Carp G.I., Reinstein J.F.R., McAlinden C. (2022). Objective and Subjective Quality of Vision After SMILE for High Myopia and Astigmatism. J. Refract. Surg..

[10] Jabbarvand M., Khodaparast M., Moravvej Z., Shahraki K., Ahmadi H., Shahraki K., Jamali A., Narooie-Noori F. (2022). Vector analysis of moderate to high myopic astigmatism after small-incision lenticule extraction (SMILE): 12-month follow-up. Eur. J. Ophthalmol..

[11] Lee J.K., Chuck R.S., Park C.Y. (2015). Femtosecond laser refractive surgery: Small-incision lenticule extraction vs. femtosecond laser-assisted LASIK. Curr. Opin. Ophthalmol..

[12] Alió Del Barrio J.L., Vargas V., Al-Shymali O., Alió J.L. (2017). Small incision lenticule extraction (SMILE) in the correction of myopic astigmatism: Outcomes and limitations—An update. Eye Vis..

[13] Zhong Y., Li M., Han T., Fu D., Zhou X. (2021). Four-year outcomes of small incision lenticule extraction (SMILE) to correct high myopic astigmatism. Br. J. Ophthalmol..

[14] Zhang Y., Shen Q., Jia Y., Zhou D., Zhou J. (2016). Clinical Outcomes of SMILE and FS-LASIK Used to Treat Myopia: A Meta-analysis. J. Refract. Surg..

[15] Song J., Cao H., Chen X., Zhao X., Zhang J., Wu G., Wang Y. (2023). Small Incision Lenticule Extraction (SMILE) Versus Laser Assisted Stromal In Situ Keratomileusis (LASIK) for Astigmatism Corrections: A Systematic Review and Meta-analysis. Am. J. Ophthalmol..

[16] Hashemi H., Asgari S., Khabazkhoob M., Heidari Z. (2023). Vector analysis of astigmatism correction after PRK, FS-LASIK, and SMILE for myopic astigmatism. Int. Ophthalmol..

[17] Zhao X., Zhang L., Ma J., Li M., Zhang J., Zhao X., Wang Y. (2021). Comparison of Wavefront-Guided Femtosecond LASIK and Optimized SMILE for Correction of Moderate-to-High Astigmatism. J. Refract. Surg..

[18] Tian H., Gao W., Xu C., Wang Y. (2023). Clinical outcomes and higher order aberrations of wavefront-guided LASIK versus SMILE for correction of myopia: A systemic review and meta-analysis. Acta Ophthalmol..

[19] Xu Y., Han Y., Lv X., Li J., Zhai C., Zhang F. (2024). Associations of Near Work, Time Outdoors, and Sleep Duration with Myopic Regression 5 Years after SMILE and FS-LASIK: A Cross-sectional Study. J. Refract. Surg..

[20] Liu J., Wang Y. (2020). Influence of Preoperative Keratometry on Refractive Outcomes for Myopia Correction with Small Incision Lenticule Extraction. J. Refract. Surg..

[21] Wang Y., Ma J. (2019). Future Developments in SMILE: Higher Degree of Myopia and Hyperopia. Asia Pac. J. Ophthalmol..

[22] Saad A., Klabe K., Kirca M., Kretz F.A.T., Auffarth G., Breyer D.R.H. (2024). Refractive outcomes of small lenticule extraction (SMILE) Pro^®^ with a 2 MHz femtosecond laser. Int. Ophthalmol..

[23] Reinstein D.Z., Archer T.J., Potter J.G., Gupta R., Wiltfang R. (2023). Refractive and Visual Outcomes of SMILE for Compound Myopic Astigmatism with the VISUMAX 800. J. Refract. Surg..

[24] Brar S., Ganesh S., Bhargav S. (2023). Comparison of Intraoperative Time Taken for Docking, Lenticule Dissection, and Overall Workflow for SMILE Performed with the VisuMax 800 versus the VisuMax 500 Femtosecond Laser. J. Refract. Surg..

[25] Reinstein D.Z., Archer T.J., Vida R.S., Carp G.I., Reinstein J.F.R., McChesney T., Potter J.G. (2022). Small Incision Lenticule Extraction (SMILE) for the Correction of High Myopia with Astigmatism. J. Refract. Surg..

[26] Chuckpaiwong V., Chansue E., Lekhanont K., Tanehsakdi M., Jongkhajornpong P., Nonpassopon M. (2023). 12-Month Outcomes of Small Incision Lenticule Extraction with Proper Head Positioning but No Reference Marking for the Correction of Astigmatism. J. Refract. Surg..

[27] Dishler J.G., Slade S., Seifert S., Schallhorn S.C. (2020). Small-Incision Lenticule Extraction (SMILE) for the Correction of Myopia with Astigmatism: Outcomes of the United States Food and Drug Administration Premarket Approval Clinical Trial. Ophthalmology.

[28] Fernández J., Valero A., Martínez J., Piñero D.P., Rodríguez-Vallejo M. (2017). Short-term outcomes of small-incision lenticule extraction (SMILE) for low, medium, and high myopia. Eur. J. Ophthalmol..

[29] Qian Y., Chen X., Naidu R.K., Zhou X. (2020). Comparison of efficacy and visual outcomes after SMILE and FS-LASIK for the correction of high myopia with the sum of myopia and astigmatism from −10.00 to −14.00 dioptres. Acta Ophthalmol..

[30] Liu H.Q., Shi J.P., Ma C.R., Zhang H., Ma X.L., Jin Y., Jin X.H., Wang H.L. (2003). [Multifactor analysis of the reasons causing undercorrection after laser in situ keratomileusis]. Zhonghua Liu Xing Bing Xue Za Zhi.

[31] Yang B., Chen J., Wang Z. (1998). Enhancement ablation for the treatment of undercorrection after excimer laser in situ keratomileusis for correcting myopia. Chin. Med. J..

[32] Moshirfar M., Jehangir N., Fenzl C.R., McCaughey M. (2017). LASIK Enhancement: Clinical and Surgical Management. J. Refract. Surg..

[33] Mohammadi S.F., Nabovati P., Mirzajani A., Ashrafi E., Vakilian B. (2015). Risk factors of regression and undercorrection in photorefractive keratectomy: A case-control study. Int. J. Ophthalmol..

[34] Lee C.Y., Jeng Y.T., Chao C.C., Lian I.B., Huang J.Y., Yang S.F., Chang C.K. (2024). Refraction and topographic risk factors for early myopic regression after small-incision lenticule extraction surgery. Sci. Rep..

[35] Chow S.S.W., Chow L.L.W., Lee C.Z., Chan T.C.Y. (2019). Astigmatism Correction Using SMILE. Asia Pac. J. Ophthalmol..

[36] Piñero D.P., Teus M.A. (2016). Clinical outcomes of small-incision lenticule extraction and femtosecond laser-assisted wavefront-guided laser in situ keratomileusis. J. Cataract. Refract. Surg..

[37] Zhang J., Wang Y., Chen X. (2016). Comparison of Moderate- to High-Astigmatism Corrections Using WaveFront-Guided Laser In Situ Keratomileusis and Small-Incision Lenticule Extraction. Cornea.

[38] Kim J., Choi S.H., Lim D.H., Yang C.M., Yoon G.J., Chung T.Y. (2019). Topography-guided versus wavefront-optimized laser in situ keratomileusis for myopia: Surgical outcomes. J. Cataract. Refract. Surg..

[39] Yoo T.K., Kim D., Kim J.S., Kim H.S., Ryu I.H., Lee I.S., Kim J.K., Na K.H. (2024). Comparison of early visual outcomes after SMILE using VISUMAX 800 and VISUMAX 500 for myopia: A retrospective matched case-control study. Sci. Rep..

